# Supplementary figures and images for: What impact has the NIHR Academic Clinical Fellowship (ACF) scheme had on clinical academic careers in England over the last 10 years? A retrospective study
Source: BMJ Open. 2017 Jun 12;7(6):e015722. doi: 10.1136/bmjopen-2016-015722 (PMC5541626; doi:10.1136/bmjopen-2016-015722)

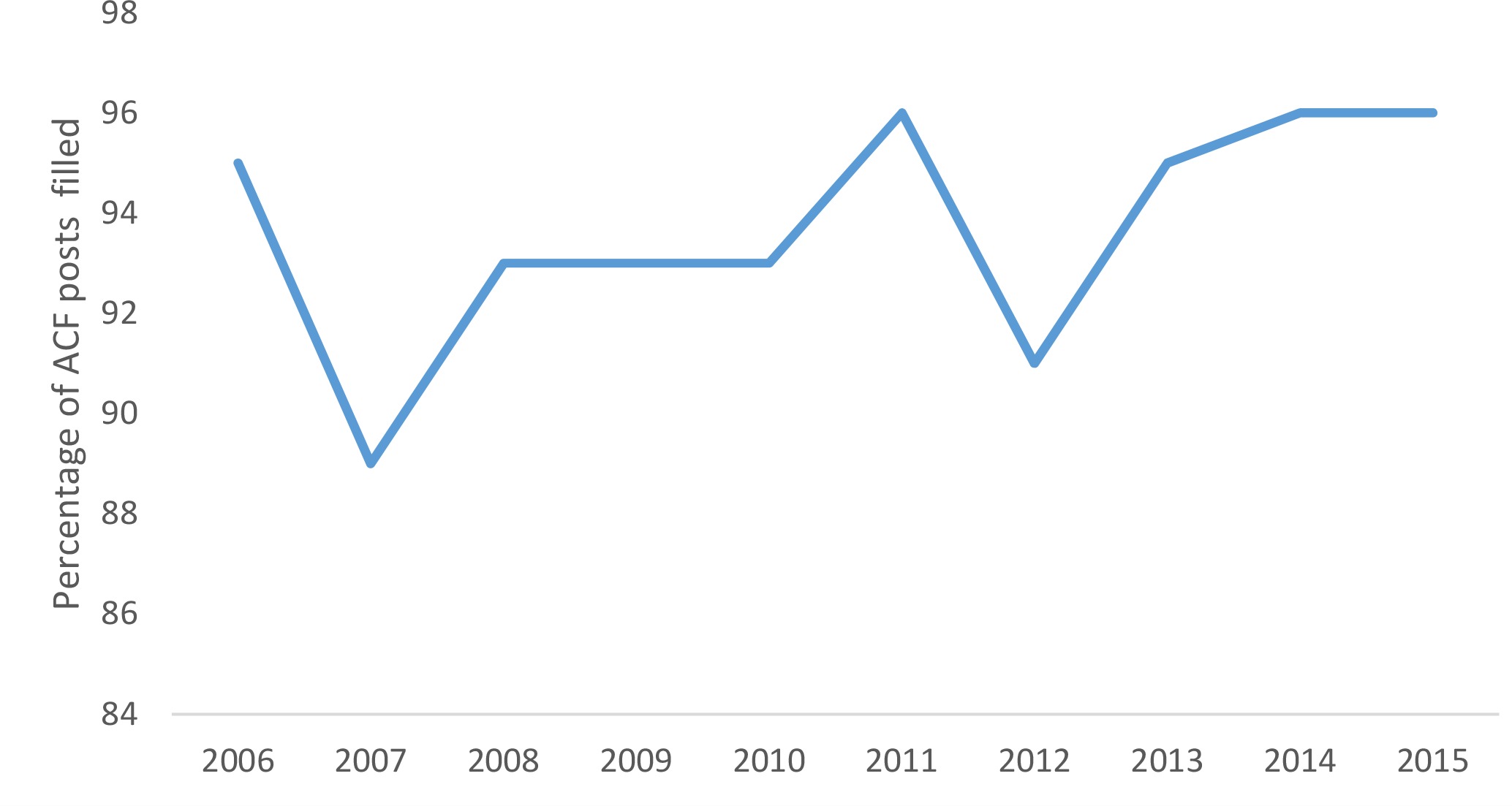

Supplement: Supplementary data [file bmjopen-2016-015722supp001.jpg]
